# Explorative Learning and Functional Inferences on a Five-Step Means-Means-End Problem in Goffin’s Cockatoos (* Cacatua goffini *)

**DOI:** 10.1371/journal.pone.0068979

**Published:** 2013-07-03

**Authors:** Alice M. I. Auersperg, Alex Kacelnik, Auguste M. P. von Bayern

**Affiliations:** 1 Department of Cognitive Biology, University of Vienna, Vienna, Austria; 2 Department of Zoology, University of Oxford, Oxford, United Kingdom; 3 Max-Planck-Institute for Ornithology, Seewiesen, Germany; University of Sheffield, United Kingdom

## Abstract

To investigate cognitive operations underlying sequential problem solving, we confronted ten Goffin’s cockatoos with a baited box locked by five different inter-locking devices. Subjects were either naïve or had watched a conspecific demonstration, and either faced all devices at once or incrementally. One naïve subject solved the problem without demonstration and with all locks present within the first five sessions (each consisting of one trial of up to 20 minutes), while five others did so after social demonstrations or incremental experience. Performance was aided by species-specific traits including neophilia, a haptic modality and persistence. Most birds showed a ratchet-like progress, rarely failing to solve a stage once they had done it once. In most transfer tests subjects reacted flexibly and sensitively to alterations of the locks’ sequencing and functionality, as expected from the presence of predictive inferences about mechanical interactions between the locks.

## Introduction

Mapping the cognitive operations by which different species solve complex problems is a central challenge to comparative cognition. Here we focus on innovation, understood as solving problems not sufficiently prevalent in a species’ ecology so as to be dealt with by narrow-domain dedicated rules shaped by natural selection, and complex enough to be difficult to resolve by unspecific, broad-domain learning rules.

In broad-domain learning processes, when a subject emits a wide variety of actions, those followed by biologically desirable consequences increase in frequency [1]. Further, because independently acquired actions may produce outcomes that constitute stimuli capable of triggering other actions, innovative concatenations of behaviour can emerge. This was exemplified by Epstein et al.’s [2] demonstration that pigeons pre-trained in individual tasks generate novel, functional sequences of up to four steps that look insightful. Thus, acquisition by piecemeal reinforcement works well if each step is either independently reinforced (as in Epstein et al.’s procedure) or added incrementally from the goal backwards, so that actions closer to the goal act as secondary reinforcements in the acquisition of actions distal to them. However, if a multiple step problem is presented without such pre-experience, downstream actions do not initially have reinforcement value and cannot acquire it because access to the goal requires earlier steps. This difficulty could be attenuated by goal representation, if the chance discovery of chain-shortening actions at the distal end are already reinforcing even if they do not directly result in reaching the goal (e.g. if moving closer to reaching the goal by effecting a distal step is reinforcing because it is perceived as progress).

After a subject has learned a sequential task through reinforcement, it is difficult to determine whether that subject has merely learnt to run through a sequence of routinized action rules triggered by the sight of certain problem features (e.g. the most distal problem always has to be attended to first regardless of its relevance for impeding the path towards the reward) or whether it has also picked up on certain functional properties of the problem. Transfer tasks testing the subjects’ sensitivity to task modifications can reveal the underlying cognitive processes [3–5].

Outside humans, whilst rare, there are documented examples of sudden innovation in multi-step problems, most notably among large-brained species such as great apes and corvids e.g. [6–19] (with few exceptions, eg. ‘chaining’ in pigeons see above 2). However, even the most complex sequences rarely exceeded three steps and mostly involved replications of similar ones. Such experiments were either based on an ‘artificial fruit’ containing food blocked by several locking devices with two or more opening mechanisms [8,18,19] or on tasks involving several tools in temporal and spatial proximity [9–16].

In the comparatively large-brained psittaciformes means-means-end (multi step means-end) problem solving has been documented in the kea, a highly explorative and neophilic New Zealand parrot that can create tool composites [17] and solve arrangements of three to four multi-step artificial fruit tasks either through social cues or after being previously trained to solve fragments of the problem [18,19]. Another species that is similarly explorative and playful is the Goffin’s cockatoo (referred to as ‘Goffin’ in future text) [20]. As is common in parrots, the Goffins’ feeding technique involves complex coordination of mandibles and a strong muscular tongue. These traits are expressed during exploratory activities [20,21]. In captivity, they have shown the capacity for innovative tool making and tool use [22].

Here we exposed Goffins to a novel five-step means-means-end task based on a sequence of multiple locking devices blocking one another. After acquisition, we exposed the cockatoos to modifications of the task such as reordering of the lock order, removal of one or more locks, or alterations of the functionality of one or more locks. Our aim was to investigate innovative problem solving under controlled conditions, to explore the mechanism of learning, and to advance towards identifying what it is that animals learn when they master a complex new sequential task.

## Materials and Methods

### Subjects

Eight juvenile (three females, five males) and two subadult (males) Goffins, all hand-raised, participated. They were housed in an enriched group aviary (14 individuals; indoors: 45m^22^ ground space, 3-6m high wall to gable; outdoors: 150 m^22^ ground space, 3-4, 5m high). Various fresh food and water sources were available *ad libitum*.

All animals that participated in the study derive from accredited European breeders, have full CITES certificates and are officially registered (following the Austrian Animal Protection Act § 25 - TschG. BGBl. 118) at the district’s administrative animal welfare bureau (Bezirkshauptmannschaft St. Pölten Schmiedgasse 4-6, A-3100; St. Pölten, Austria). The described housing conditions are in accordance with the species specific guidelines provided by the Austrian Federal Act on the Protection of Animals (Animal Protection Act -§ 24 Abs. 1 Z 1 and 2; § 25 Abs. 3 – TSchG, BGBl. I Nr. 118/2004 Art. 2). Furthermore, as our experiments are purely appetitive, strictly non-invasive and based exclusively on behavioural tests, they are classified as non-animal experiments in accordance with the Austrian Animal Experiments Act (§ 2. Federal Law Gazette No. 501/1989). Our animals are not clipped and participation in all experiments occurs principally on a voluntary basis: either the door of the testing compartment is opened and the respective bird is called by name, or the experimenter enters the group space and asks the subject to step up on his/her hand in order to be carried into the testing chamber.

None of the animals had experimental history or experience related to the present context (animals had at the time only participated in one experiment on Piagetian object permanence [23]). For the acquisition phase, all 14 subjects were initially randomly assigned to four groups (described in detail below under procedures): Individual Simultaneous (**IndSim**), Individual Incremental (**IndInc**), Social Simultaneous (**SocSim**) and Social Incremental (**SocInc**). However, four subjects could not be tested because they did not habituate to the experimental compartment. Therefore the final numbers were four subjects in the **IndSim** group and two in each of the other groups.

### Apparatus

**Figure 1:**
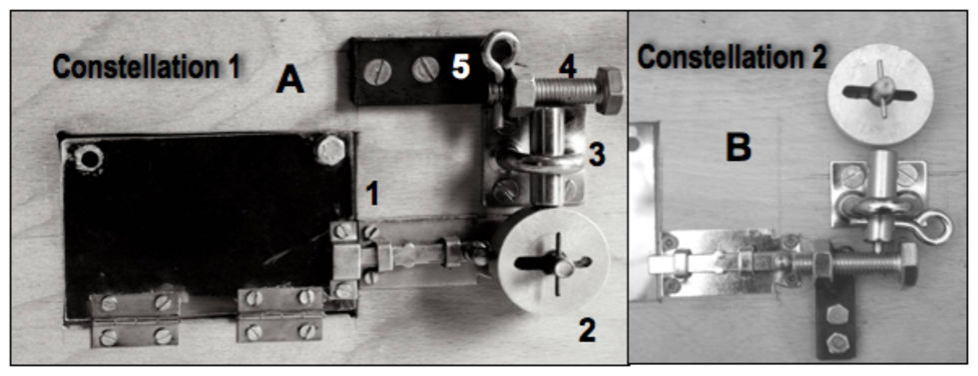
A & B. Basic configurations. *A*) basic task configuration for acquisition & transfer test 1, 3 and 4. L5) pin; L4) screw; L3) bolt; L2) wheel; L1) bar. The pin is inserted through a perforation in the screw end; the screw is held by a fixed nut and blocks the upward movement of the bolt; a protrusion in the bolt’s end fits into a recess on the wheel’s edge, blocking its rotation; the wheel impedes the displacement of the bar, which blocks the window behind which is the food. To be removed, the wheel has to be rotated to align its central slot to the T-bar passing through its axis. *B*) configuration for TT 2. The position of all locks except ‘bar’ has been altered (a recess in the screw matched the bolt’s protrusion, and a passing hole through the bolt let the pin go through so that the pin had to be removed for the bolt to be lifted).

**Figure 2:**
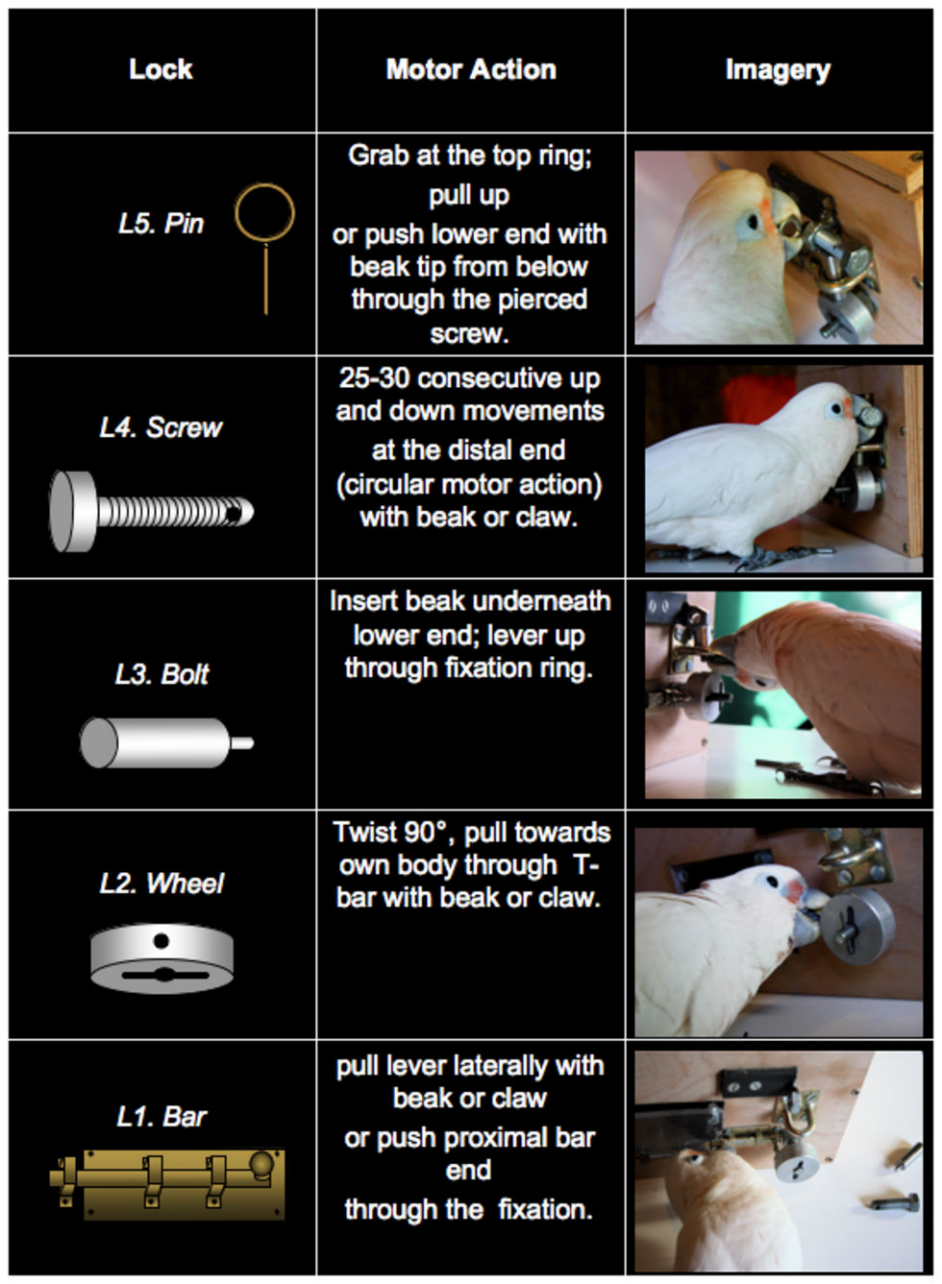
Actions required for removing each individual lock (L1-L5).

The apparatus consisted of a wooden box (25x15x15 cm; Figure 1 A) with a transparent, acrylic window at its front (10x7 cm). Behind this window a food reward (1/4 cashew nut) was visibly displayed. Opening of the window to retrieve the food was impeded by five locks labeled L1 to L5 from the nearest to the most distant to the goal. In all experiments the window was blocked with a flat bar (L1). From then on the configuration varied according to condition, but the original configuration was as follows. The bar (L1), a wheel (L2), a cylindrical bolt (L3), a screw (L4) and a pin (L5; see Figure 1 A for details). In this basic configuration the required sequence was L5 → L4 → L3 → L2 → L1 → GOAL. Note that the removal of each lock required different actions that could be (and were) tackled differently by different individuals (Figure 2).

### Procedure

Testing was conducted on a table (1x1m) in a visually isolated compartment in the presence of an experimenter (Constanze Riha or AA). The experimenter sat on a chair facing the apparatus (observing the events from behind the animal) to prevent unintended cueing. Additionally, the experimenter wore mirrored sunglasses during the transfer tests. Data were collected throughout August–November 2011.

For each of the procedures described below, subjects received up to five sessions of up to ten trials, with trials lasting up to 20 min. If subjects retrieved the reward in the first trial of a session, they received up to nine further trials within the same session. If they failed to reach the reward within 20 min within one trial, the session was terminated and they received a new session on the following day. The experiment advanced to the next phase once the birds had retrieved the food for two consecutive sessions of ten trials each in the training of all subjects and later in two of the groups (**IndSim & SocSim**). In the Incremental groups (**SocInc & IndInc**) subjects received the next step (i.e. a new lock was added) after a session of ten successful trials with the previous device. If birds did not succeed to reach the goal within five sessions, the procedure that followed differed among groups (see below).

During the pre-training (just L1, the bar) and the task acquisition phase (i.e. not during transfer tests), subjects occasionally directed their attention away from the apparatus. When this happened the experimenter (Constanze Riha) was allowed to tap the wood of the box (not the locks) and/or placed the subject back in front. If subjects started to manipulate detached locks after they had been removed from the apparatus, the experimenter removed them.

#### Familiarization and pre-training phase

All subjects first received pre-training in which just the bar (L1) was present. Birds in the ‘individual’ groups were exposed to the box with no other bird present. Those in the ‘social’ groups received three demonstrations by a conspecific (demonstrators were subjects from other groups that had already accomplished the full task) at the beginning of each new session (see below). Subjects received five sessions of up to ten trials. In order to obtain more subjects for transfer tests, unsuccessful subjects from the ‘individual’ groups could receive additional sessions preceded by demonstrations, while subjects that were unsuccessful despite social demonstrations did not proceed to testing.

#### Task acquisition phase

1) Individual Simultaneous.

After the pre-training, four birds were directly confronted with all five locks, in social isolation.

So as to have subjects for later transfer tasks, birds that failed the **IndSim** condition still had the chance to solve the task in an incremental manner and/or with social cues; in this case, they first faced a combination of two locks (L1 & L2) for a maximum of five sessions. Upon success, the combination was changed back to the original five locks for one session. If they could not solve the five locks condition, they were presented with three locks (L1-3). This continued in a stepwise, progressive lengthening of the sequence. Those that still failed to open certain configurations for more than five sessions, received further extra trials with social demonstrations (i.e. were treated as the **SocInc** group, see below) for that particular lock.

2) Individual Incremental.

Following pre-training, the two subjects in this group received L1 combined with L2 (step2) and were given five sessions to solve it. The number of locks was increased progressively (steps 3-5) with the same procedure, continuing from L1, L2 and L3 until the birds removed all five locks in the sequence (step 5 = the test situation of the **IndSim** group). However since both subjects failed the group conditions, being stuck on one of the locks (five consecutive unsuccessful sessions), they received additional trials with social demonstrations (see **SocSim** and **SocInc** for the procedure) for that particular step.

3) Social Simultaneous.

The two subjects in this group received three demonstrations by a conspecific that previously had been in one of the 'individual' groups (we chose demonstrators that were tolerant to/compatible with the subject, see File S1) solving the entire sequence of locks prior to each trial. Again, as both failed the criteria of the **SocSim** condition they next received additional trials with incremental demonstrations (see **SocInc** procedure).

4) Social Incremental.

The two subjects in this group were presented with all locks in a stepwise manner starting with L1 & L2 in the first session. Prior to each session of a new step (and until first success in that respective step) they received three demonstrations from a conspecific. Both subjects failed this group’s requirements and were removed from the experiment.

#### Transfer Tests

The 6 (out of 10) subjects that succeeded in removing all locks in the task acquisition phase proceeded to transfer tasks. Transfer tests were transformations of the original configuration designed to expose the birds’ response to the functionality of each lock in the chain.

1) Transfer Test 1 (TT1): one lock missing.

**Figure 3:**
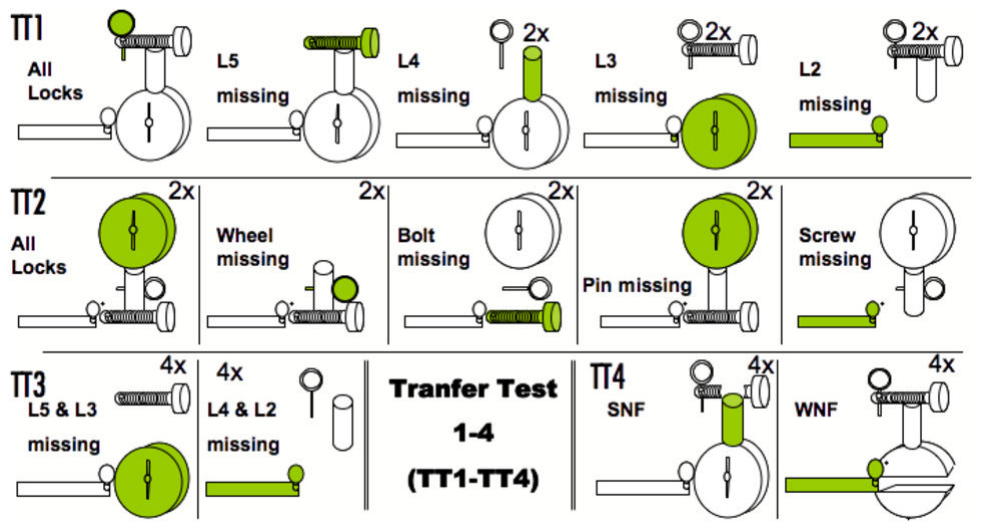
Transfer Tests 1-4. In TT1 and TT3 one or two locks were removed; in TT2 both configuration and order of tasks were changed as well as removing one or no locks. In TT4 either the wheel (WNF) or the screw (SNF) were non-functional but their edges were still touching the lower and upper locks (2x or 4x = no. of times each condition was presented in each session of ten trials). Subjects received two sessions of TT1 and TT2 and one session of TT3 and TT4. Correct behaviour was scored when the bird touched and removed first the lock marked green.

TT1 tested whether the birds had developed a rigid behavioral routine running through the locks from the most distal to the most proximal device, or focused directly on the first (effective) lock after a chain interruption. We had five possible conditions, the original one plus four conditions with one of the locks missing (Figure 3; when necessary due to the absence of the screw, the pin was fixed in its original position using transparent adhesive tape; this was also used to fix the pin in absence of the screw or the bolt in TT2 and TT3). Each condition was presented twice per session of ten trials (in random order). Since we were only interested in performance without much opportunity to re-learn, each individual received just two sessions, hence each condition four times. We counted, conservatively, the first lock touched by a subject as either correct (if it was the first lock downstream from the missing one) or incorrect (any other lock).

2) Transfer Test 2 (TT2): new configuration, one lock missing.

Birds that were successful in TT1 could hypothetically also go for a partial setup looking similar to one they had previously operated in the acquisition phase. Therefore, TT2 maintained the idea of removing part of the sequence, but the order of the locks was additionally scrambled (Figure 1B, Figure 3). Here, a fully functional response would have to cope at once with both the alteration of the lock order and the occasional removal of one of them. As before, each condition occurred twice in each session and subjects received two sessions.

3) Transfer test 3 (TT3): two locks missing.

Success in TT1 and TT2 could be due to routinely dealing first with any ‘open end’, namely any lock not immobilized, disregarding its role in blocking the chain. To test whether this is how the birds did it, we used the original configuration with two missing locks (Figure 3): subjects received one session of ten trials, four in which L5 and L3 was missing, and four in which L4 and L2 were missing, (and two intermission reminders with the original configuration), in random order. Trials were rated as correct if subjects touched first the lock downstream from where the missing lock closer to the goal was.

4) Transfer test 4 (TT4): one non-functional lock (screw or wheel).

TT4 explored sensitivity to the functional integrity of the locks. Birds could have solved TT1-TT3 by starting with the most distal of lock connected to the chain upstream from the goal (a continuity rule), or by paying attention to the points of connection between locks, in both cases without attending to the physical way by which each lock blocked the following one. In either case they would tackle a lock that was part of the chain even if this was no longer necessary due to a structural change. We designed two configurations in which either the wheel or the screw had a middle section cut out, so that they were non-functional (i.e. not effectively blocking), whilst their edges were still touching the sides of the previous and next lock (We used Tesa® powerstrips to fixate the spare parts of the locks to the box; Figure 3). In this case L1 or L3 respectively could be opened without the need to remove any upstream lock. The subjects received one session of ten trials in this final condition. Each session comprised four trials of the non-functional screw condition, four trials of the non-functional wheel condition (and two intermission reminders with the original configuration), in random order.

### Analysis

All trials were videotaped. For the acquisition phase we recorded which locks each bird removed. Additionally, we measured the latency to remove each subsequent lock to the nearest five seconds. Inter-observer reliability (naïve scorer; 20% of the data) for the acquisition phase was ‘almost perfect’ according to statistic classifications (Kappa value=0.86). To examine the pattern of shortening of the latency to solve each lock as a function of experience during acquisition we conducted a Friedman’s test over the first five lock removals (Table S2 in File S1). To investigate the time-shortening between the first and subsequent removals of each lock, we ran post-hoc non-parametric pairwise statistics for dependent samples (Wilcoxon-signed rank; Table S3 in File S1).

For the transfer tests nonparametric one-sample statistics were used to test whether subjects chose the correct lock significantly more often than other locks for each of the conditions in each of the subtasks. Chance level of the correct lock being touched first was calculated as the number of trials divided by the number of locks present, namely 4/5 when none was missing, 4/4 when one was and 4/3 when two locks were missing. All tests we used were two tailed.

## Results

### Task Acquisition Phase

Progress differed between individuals, but qualitatively followed a pattern of exploration of the setup’s affordances followed by cumulative approximations to the goal: most birds attended first to the goal compartment, trying to reach the cashew by rattling the window or the bar that was directly blocking it. Next, they explored physical features of the box with their beaks and feet. In this phase they moved each lock within the margin allowed by the engagement of neighboring devices. In the basic simultaneous configuration the first device that could be removed was the pin, followed by the screw, etc.

In the **IndSim** group, one subadult male (Pipin), removed all locks and reached the goal in his fifth session (hence in less than 100 min cumulative time). He never failed to open a lock after a first success, and this also applied to the entire sequence (Table S1 in File S1). Pipin was alone in solving the problem unassisted and was also the only bird using his foot instead of his beak to remove the screw (Movie S1 in File S1). Two further members of this group eventually learned to remove all locks, but only after additional incremental exposure and social demonstrations.

Both members of the **IndInc** group opened four of the five locks within group conditions and eventually opened also the remaining one (wheel or screw), but only after additional social demonstrations.

The two subjects in the **SocSim** group only learned to remove the pin within the first five sessions. One of them later mastered the whole task after receiving additional incremental trials. The remaining one removed three locks after incremental trials but never discovered how to remove the screw.

In the **SocInc** group one cockatoo removed two locks (the bar and the wheel) within the time given and the other failed to open any locks. Neither of them proceeded to the transfer tests. A more detailed description of individual task acquisition is shown in further text in the electronic Supporting Information (see text and Table S6 in File S1).

**Figure 4:**
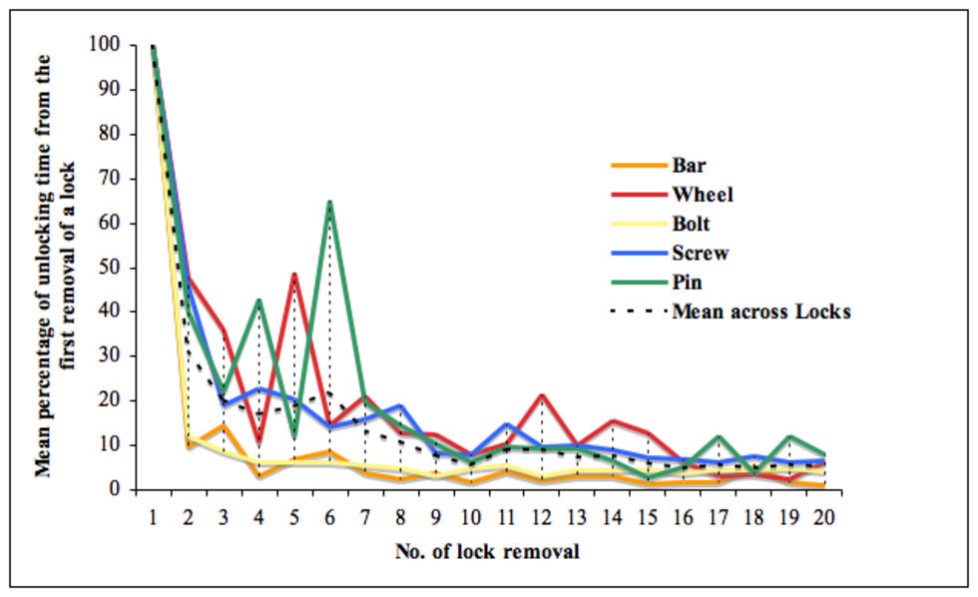
Mean percentage of time it took the animals to remove each lock. The mean percentage of time it took the animals to remove each lock was measured between completion of successive steps. Data from first 20 successful removals during acquisition phase (locks represented by the coloured lines; dashed line represents average across locks; Times are standardized so that first removal of each lock is 100%).

Notably, once the birds discovered how to remove a particular lock for the first time, they seldom (seven out of 32 cases) failed to open the same one in later trials. This means that in most cases (78%), progress was stepwise rather than gradual for individual lock removals, and this resulted in a ratchet-like progress over the whole sequence (Table S1 in File S1). The total time taken to remove a particular lock (measured from completing the previous step to completing that one) also showed a quasi-discontinuous evolution: there was an abrupt shortening of the time taken between the first and second removal of each lock, followed by a slow progressive further shortening with practice (between-subjects mean in Figure 4; for individual details see Figure S1 and Tables S2, and S3 in File S1). Qualitative improvement continued throughout the experiment: Three individuals discovered how to displace locks just sufficiently to release the catch on the following one, without having to remove them as we expected through the system’s design. They just lifted the lower end of the bolt within its holding ring to allow the wheel to rotate, and pushed out the wheel only as far as necessary to stop it from immobilizing the bar (Movie S1 in File S1).

### Transfer tests

Subjects always reached the reward in the transfer tasks.

#### TT1

**Figure 5:**
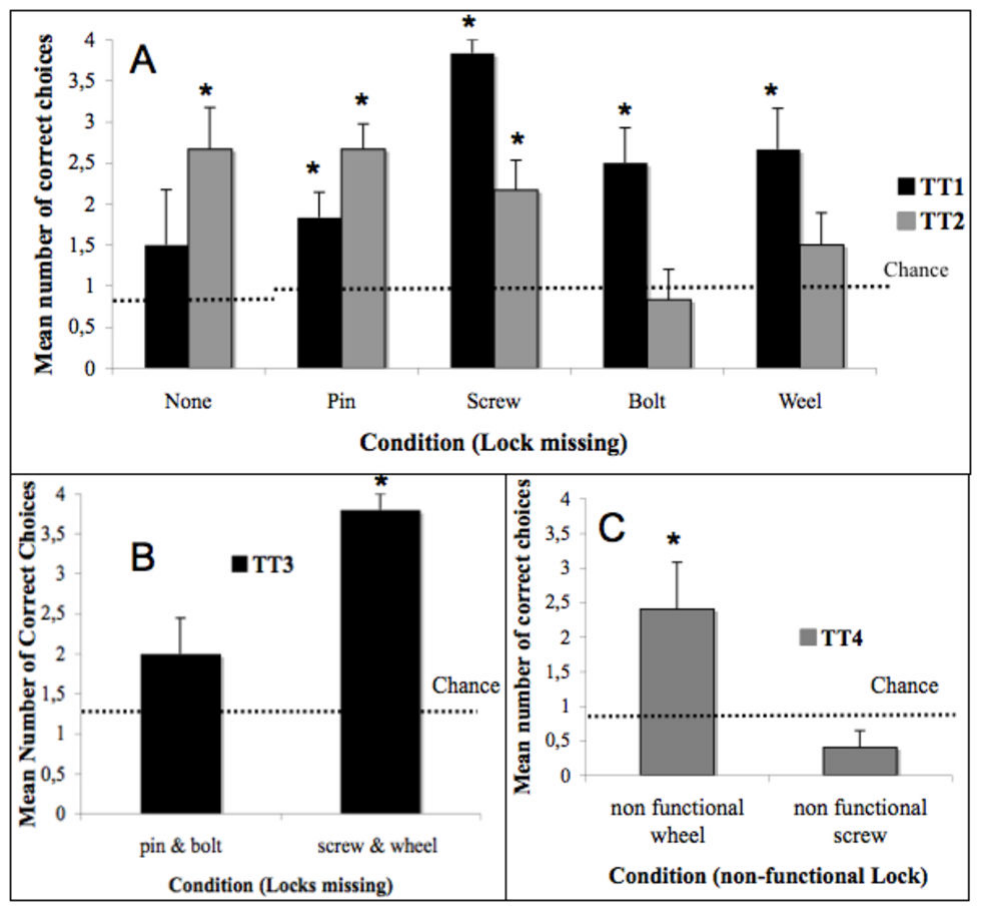
A-C. Mean number of correct choices in the transfer tasks. The mean number of correct choices in the transfer tasks (first lock touched out of four trials) and chance expectation are given for each of the conditions in the transfer tests (locks missing or non-functional); A) TT1 (black) and TT2 (grey) B) TT3; C) TT4. * Number of correct choices differed significantly from chance (two tailed).

In all conditions in which one lock was missing, the birds touched the correct lock first significantly above chance (Figure 5A, Table S4 in File S1). When all locks were present the animals correctly touched first the pin (i.e. the correct device) in 37.5% of the trials, against a random expectation of 20%, but this does not reach conventional statistical two-tailed significance.

#### TT2

Performance was not affected by scrambling the order: when all locks were present they correctly touched the new functional beginning (the wheel) first significantly above chance. They also correctly identified the first functional device in two of the discontinuity tasks; when either the pin or the screw was missing but not when the wheel or the bolt had been removed (Figure 5A, Table S4 in File S1).

#### TT3

The birds touched first the correct one (the bar) significantly above chance when the wheel and screw were missing (Table S5 in File S1). When the pin and bolt were missing (Figure 5B) they touched the wheel first in 45% of the trials, against a chance expectation of 33%, which was non-significant.

#### TT4

Subjects correctly addressed the bar first when the wheel had been cut, but failed to go for the bolt when the screw had been sectioned inappropriately choosing the bar in this condition as well (Figure 5C, Table S5 in File S1).

For detailed information on individual performance in transfer tests, see Table S4, S5 and Movie S2 in File S1.

## Discussion

The initial acquisition of the multiple lock problem was remarkable in that the cockatoos progressed by a combination of intense exploration and manipulation: the cockatoos interacted extensively with the apparatus before discovering partial or whole solutions, exhibiting diverse haptic exploratory behaviors. Characteristically of parrots [24,25], these involved bill, tongue and feet, permitting a greater diversity of actions than that of birds employing their beak only. Subjects were highly persistent, and the haptic nature of their exploration may have given them a crucial advantage, as it is likely that a purely visual explorer would never have detected the necessary affordances.

Prior to this study, the birds had never been systematically taught to remove objects for obtaining a reward. Yet, one subject solved the entire problem unassisted in five sessions and never failed to repeat a successful manipulation. Pipin’s acquisition performance is highly innovative, but consistent with established principles of learning by consequences provided that goal-directed exploration is factored in [4]. Interestingly, his rapid route to perfection in later trials indicates that the complex sequence of different motor actions required for opening the entire arrangement was added to a recallable behavioral toolkit almost instantly after each success.

To our knowledge, except for field reports of tool sets in wild chimpanzees [26,27], sequential problem solving by non-humans involving more than three different steps, without prior training by shaping, as in Pipin’s case has never been reported; in sequential tool-use tasks apes [28] have been reported to master up to five steps, and New Caledonian crows three [12,13]. However, in the present study, each step required several different sensorimotor actions, while in other sequential tasks intermediate actions were close to being repetitions of previous ones, e.g. using a short tool to retrieve a medium length tool to retrieve a long enough tool, so that each action by itself was not new.

Seven other birds eventually opened part of the sequence, and five of the latter solved the whole chain after being exposed to the sequence incrementally, letting them observe a skilled conspecific, or both. The fact that most individuals solving the entire problem did so after additional social or incremental scaffolding suggests that the two latter factors may be influential. However, since most birds (except for Pipin) did not meet their initial group requirements and due to the strong differences in performance between individuals, we cannot presently disentangle these factors from longer exposure to the apparatus.

The ‘assisted’ subjects, nevertheless, also showed an almost perfect ability to replicate individual lock removals once they had succeeded once. Such ability to quickly repeat successful actions does not necessarily have to be uncharacteristic of reinforcement learning [29]. However, since the removal of some individual locks (e.g. bolt, wheel & screw) did in itself require a distinct set of spatio-temporally adjusted motor actions, the birds’ sudden improvement in removing them does indicate pronounced levels of behavioral plasticity, sensorimotor control and practical memory in this species.

The behavior of three individuals, which displaced some of the locks just enough to be able to move the following ones without having to remove them entirely further implies that the animals developed sensitivity towards the blocking effect of the locks during the acquisition phase. The majority of the results of the Transfer Tests further support this idea: when one lock was missing, they directed their attention to the first relevant one (surprisingly this was significant with the exception of the condition in which no locks were missing), ruling out a rigid learned routine. Their performance remained above chance in most conditions when the lock arrangement was scrambled, eliminating the possibility that the birds simply learned to open the locks in a given succession. In the two conditions in which two non-adjacent locks were removed, the animals’ performance was conventionally significant in one case and marginally so in the other. The data can be interpreted to suggest that they did not simply resort to memories of how any ‘movable’ lock looked regardless of its blocking properties.

The last task exposed the birds to locks that had been made ineffective by removal of the middle section. The birds appropriately chose the bar when the wheel was ineffective. However, the birds ignored the ineffective screw, but also touched first the bar, which was not correct. The latter behaviour could be a residual effect from the pre-training in which all subjects faced only the bar. The results however render it unlikely that the birds used a connectivity rule (to always start at the distal end of a connected sequence of locks towards the goal).

The proclivity of different species to produce novel functional behavior is a core topic in cognitive research, because innovation challenges most currently available explicit algorithms describing processes of behavior modification [24,30,31]. The Goffins’, in particular the subject Pipin’s, ability to solve a complex five-step means-means-end task reveals an innovative capacity beyond that reported so far in other species of birds and most mammals. Nevertheless, our study also highlights the importance of parsimony in the interpretation of physical cognition results. The main challenge of cognitive research is to map the processes by which animals gather and use information to come up with innovative solutions to novel problems [32–34], and this is not achieved by invoking mentalistic concepts as explanations for complex behaviour. Dissecting the subjects’ performance to expose their path towards the solution and their response to task modifications can be productive; even extraordinary demonstrations of innovative capacity are not proof of the involvement of high-level mental faculties, and conversely, high levels of cognition could be involved in seemingly simple tasks. The findings from the transfer tests allow us to evaluate some of the cognition behind the Goffins’ behaviour. Although the exact processes still remain only partially understood, our results largely support the supposition that subjects learn by combining intense exploratory behavior, learning from consequences, and some sense of goal directedness. This combination allows them to act flexibly and sensitively to the functional properties of the task rather than running through learned sequences in inflexible, fixed-rule-governed manners.

## Supporting Information

File S1 Summarized supporting text, tables (Tables S1-S6), movie links (Movies S1 & S2) and a figure (Figure S1).
(DOC)
