# Supplementary material for: Explorative Learning and Functional Inferences on a Five-Step Means-Means-End Problem in Goffin’s Cockatoos ( Cacatua goffini )
Source: PLoS One. 2013 Jul 3;8(7):e68979. doi: 10.1371/journal.pone.0068979 (PMC3700958; doi:10.1371/journal.pone.0068979)
Supplement: File S1 — Summarized supporting text, tables (Tables S1-S6), movie links (Movies S1 & S2) and a figure (Figure S1). (DOC) [file pone.0068979.s001.doc]

**SUPPORTING MATERIAL**

**Explorative learning and functional inferences on a five-step means-end problem in Goffin’s cockatoos (*Cacatua goffini*)**

Alice M.I. Auersperg, Alex Kacelnik and Auguste M.P. von Bayern

**Supporting Details of the Methodology:**

**Demonstrators:**

Note that all subjects from the ‘Individual’ groups except for Pipin received demonstrations for one or two of the locks after failing the initial group requirements.

*Subject 🡪 Demonstrator*

Pipin (IndSim) 🡪  None

Heidi (IndSim) 🡪  Figaro

Moneypenny (IndSim) 🡪 Pipin

Muppet (SocSim)🡪 Konrad

Figaro (SocSim)🡪 Konrad

Dolittle (IndInc) 🡪  Konrad

Konrad (InInc) 🡪  Pipin

Mayday (SocInc)🡪 Dolittle

Kiwi (SocInc) 🡪 Konrad

**Movie S1:** Video examples of the original configuration:

<http://www.zoo.ox.ac.uk/group/kacelnik/lb_movie_s1.mov>

1) Complete lock removal. 2) Example of unlocking the screw with the claw. 3) Example of partial lock removal (L2 and L3).

**Movie S2:** Video examples of the Transfer Tasks.

<http://www.zoo.ox.ac.uk/group/kacelnik/lb_movie_s2.mov>

1) TT1: screw missing. 2) TT1: bolt missing. 3) TT2: none missing; 4) TT2: bolt missing. 5) TT3: screw & wheel missing. 6) TT4: pin and bolt missing. 7) TT4: non-functional screw. T8) TT5: non-functional wheel.

**Supporting Results**

1. ***Detailed results for the task acquisition phase***

**Table S1. Failures to re-open each lock following the first unlocking success during the task acquisition phase.**

**Failures to re-open each lock (bar, wheel, bolt, screw, pin) in the trials following the first unlocking success during the task acquisition phase. *Right*: Proportion of devices for which there were no failures after a first success.**

| **Subject** | *bar* | *wheel* | *bolt* | *screw* | *pin* | **Prop. of locks**  **no failures** |
| --- | --- | --- | --- | --- | --- | --- |
| *Dolittle* | 3 | 8 | 0 | 0 | 0 | 0,6 |
| *Heidi* | 0 | 0 | 0 | 5 | 0 | 0,8 |
| *Konrad* | 0 | 0 | 0 | 0 | 0 | 1 |
| *Moneypenny* | 0 | 2 | 0 | 3 | 0 | 0,6 |
| *Muppet* | 2 | 0 | 0 | 0 | 0 | 0,8 |
| *Pipin* | 0 | 0 | 0 | 0 | 0 | 1 |
| *Figaro* | 0 | 0 | 0 |  |  | 1 |
| *Mayday* | 0 | 3 |  |  |  | 0,5 |
|  | | | | | **Mean** | 0,79 |
|  |  |  |  |  | **SD** | 0,189 |
|  |  |  |  |  | **SE** | 0,067 |

**Figure S1.**


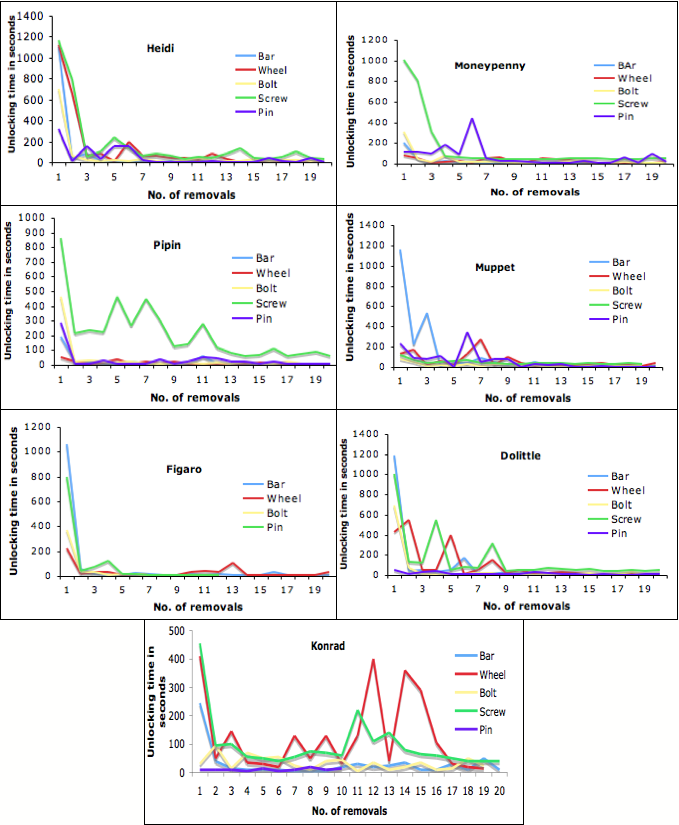


**Figure S1.** **Unlocking time for the 20 first successful removals of each lock for each subject.** Unlocking time in seconds for each lock (Bar-Pin) for individual subjects over the first 20 successful removals of the respective locks during the task acquisition phase (trials in which a lock was not opened are excluded).

**Table S2. Outcome Friedman’s ANOVA over the first five removals (R1-R5) for each of the five locks.**

(Pin-Bar) during task acquisition. Significant outcomes are bold framed (all p-values are above chance expectation).

| **Locks** | **Medium Ranks** | | | | | ***N, df*** | ***X^2^*** | ***p-value*** |
| --- | --- | --- | --- | --- | --- | --- | --- | --- |
|  | ***R1*** | ***R2*** | ***R3*** | ***R4*** | ***R5*** |  |  |  |
| *Pin* | 4.5 | 2.29 | 2.64 | 3.43 | 2.14 | 7,4 | 11.134 | 0.025 |
| *Screw* | 5 | 3 | 2.6 | 2.4 | 2 | 5,4 | 11.04 | 0.026 |
| *Bolt* | 5 | 3,36 | 3 | 1.86 | 1.79 | 7,4 | 19.623 | 0.001 |
| *Wheel* | 4.71 | 3.71 | 2 | 2.43 | 2.14 | 7,4 | 15.652 | 0.004 |
| *Bar* | 5 | 3.38 | 2.88 | 1.75 | 2 | 8,4 | 22.051 | 0.0001 |

**Table S3. Post-hoc-paired tests between the first five removals (R1-R5) for each lock (Pin-Bar) during task acquisition.**

Significant outcomes (p<0.05) are bold framed.

| **Locks** | **Lock Removal** | **Z-Value** | **N;df** | **p-Value** |
| --- | --- | --- | --- | --- |
| *Pin* | R 1-R2 | 2.023 | 7;5 | 0.043 |
|  | R1 -R3 | 2.201 |  | 0.028 |
|  | R1-R4 | 1.859 |  | 0.063 |
|  | R1-R5 | 2.197 |  | 0.028 |
|  | R2-R3 | 1.214 |  | 0.225 |
|  | R2-R4 | 2.201 |  | 0.028 |
|  | R2-R5 | 0.511 |  | 0.61 |
|  | R3-R4 | 1.014 |  | 0.31 |
|  | R3-R5 | 1.706 |  | 0.088 |
|  | R4-R5 | 1.018 |  | 0.309 |
| *Screw* | R 1-R2 | 2.023 | 5;3 | 0.043 |
|  | R1 -R3 | 2.023 |  | 0.043 |
|  | R1-R4 | 2.023 |  | 0.043 |
|  | R1-R5 | 2.023 |  | 0.042 |
|  | R2-R3 | 0.813 |  | 0.416 |
|  | R2-R4 | 0.944 |  | 0.345 |
|  | R2-R5 | 1.214 |  | 0.225 |
|  | R3-R4 | 0.000 |  | 1 |
|  | R3-R5 | 0.135 |  | 0.893 |
|  | R4-R5 | 0.135 |  | 0.892 |
| *Bolt* | R 1-R2 | 2.366 | 7;5 | 0.018 |
|  | R1 -R3 | 2.366 |  | 0.018 |
|  | R1-R4 | 2.366 |  | 0.018 |
|  | R1-R5 | 2.371 |  | 0.018 |
|  | R2-R3 | 0.734 |  | 0.463 |
|  | R2-R4 | 1.521 |  | 0.128 |
|  | R2-R5 | 1.696 |  | 0.090 |
|  | R3-R4 | 0.947 |  | 0.344 |
|  | R3-R5 | 2.226 |  | 0.026 |
|  | R4-R5 | 0.170 |  | 0.865 |
| *Wheel* | R 1-R2 | 1.183 | 7;5 | 0.237 |
|  | R1 -R3 | 2.366 |  | 0.018 |
|  | R1-R4 | 2.366 |  | 0.018 |
|  | R1-R5 | 2.366 |  | 0.018 |
|  | R2-R3 | 1.439 |  | 0.15 |
|  | R2-R4 | 2.028 |  | 0.043 |
|  | R2-R5 | 1.917 |  | 0.051 |
|  | R3-R4 | 0.674 |  | 0.5 |
|  | R3-R5 | 0.000 |  | 1 |
|  | R4-R5 | 0.254 |  | 0.79 |
| *Bar* | R 1-R2 | 2.521 | 8;6 | 0.012 |
|  | R1 -R3 | 2.521 |  | 0.012 |
|  | R1-R4 | 2.521 |  | 0.012 |
|  | R1-R5 | 2.521 |  | 0.012 |
|  | R2-R3 | 0.421 |  | 0.674 |
|  | R2-R4 | 2.533 |  | 0.011 |
|  | R2-R5 | 1.402 |  | 0.161 |
|  | R3-R4 | 1.265 |  | 0.206 |
|  | R3-R5 | 1.156 |  | 0.248 |
|  | R4-R5 | 0.841 |  | 0.4 |

***2. Detailed results of the transfer tests***

**Table S4.** **Detailed individual data for TT1 & TT2 for each subject.**

Detailed individual data: first lock touched within the four trials of each condition (depending on the missing lock) for TT1 & TT2 for each individual subject. The correct response is highlighted in grey, and lock columns touched first above chance are bold framed.

|  |  | Lock first touched first in total four trials | | | | |
| --- | --- | --- | --- | --- | --- | --- |
| **Transfer** | **Subjects** | *Pin* | *Screw* | *Bolt* | *Wheel* | *Bar* |
| **TT 1** |  | **No Locks missing condition** | | | | |
|  | *Dolittle* | 3 | 1 | 0 | 0 | 0 |
|  | *Heidi* | 4 | 0 | 0 | 0 | 0 |
|  | *Konrad* | 0 | 2 | 0 | 0 | 2 |
|  | *Moneypenny* | 0 | 2 | 2 | 0 | 0 |
|  | *Muppet* | 1 | 0 | 3 | 0 | 1 |
|  | *Pipin* | 1 | 0 | 1 | 0 | 2 |
| **TT 2** | *Dolittle* | 0 | 0 | 0 | 4 | 0 |
|  | *Heidi* | 1 | 0 | 0 | 3 | 0 |
|  | *Konrad* | 1 | 0 | 0 | 3 | 0 |
|  | *Moneypenny* | 0 | 0 | 0 | 4 | 0 |
|  | *Muppet* | 2 | 0 | 1 | 1 | 0 |
|  | *Pipin* | 1 | 0 | 0 | 1 | 2 |
|  |  | **Pin missing condition** | | | | |
| **TT 1** | *Dolittle* | / | 2 | 1 | 0 | 1 |
|  | *Heidi* | / | 3 | 1 | 0 | 0 |
|  | *Konrad* | / | 1 | 2 | 0 | 1 |
|  | *Moneypenny* | / | 2 | 1 | 0 | 1 |
|  | *Muppet* | / | 2 | 1 | 1 | 0 |
|  | *Pipin* | / | 1 | 1 | 0 | 1 |
| **TT 2** | *Dolittle* | / | 0 | 0 | 3 | 1 |
|  | *Heidi* | / | 0 | 0 | 4 | 0 |
|  | *Konrad* | / | 0 | 2 | 2 | 0 |
|  | *Moneypenny* | / | 0 | 1 | 3 | 0 |
|  | *Muppet* | / | 0 | 2 | 2 | 0 |
|  | *Pipin* | / | 0 | 1 | 2 | 1 |
| **TT 1** |  | **Screw missing condition** | | | | |
|  | *Dolittle* | 0 | / | 4 | 0 | 0 |
|  | *Heidi* | 0 | / | 4 | 0 | 0 |
|  | *Konrad* | 0 | / | 4 | 0 | 0 |
|  | *Moneypenny* | 0 | / | 4 | 0 | 0 |
|  | *Muppet* | 0 | / | 4 | 0 | 0 |
|  | *Pipin* | 0 | / | 3 | 0 | 1 |
| **TT 2** | *Dolittle* | 0 | / | 0 | 3 | 1 |
|  | *Heidi* | 0 | / | 0 | 2 | 2 |
|  | *Konrad* | 0 | / | 2 | 1 | 1 |
|  | *Moneypenny* | 0 | / | 0 | 1 | 3 |
|  | *Muppet* | 0 | / | 0 | 1 | 3 |
|  | *Pipin* | 0 | / | 0 | 1 | 3 |
| **TT 1** |  | **Bolt missing condition** | | | | |
|  | *Dolittle* | 2 | 0 | / | 2 | 0 |
|  | *Heidi* | 1 | 0 | / | 3 | 0 |
|  | *Konrad* | 1 | 1 | / | 1 | 1 |
|  | *Moneypenny* | 0 | 0 | / | 4 | 0 |
|  | *Muppet* | 1 | 0 | / | 3 | 0 |
|  | *Pipin* | 0 | 0 | / | 3 | 1 |
| **TT 2** | *Dolittle* | 0 | 0 | / | 4 | 0 |
|  | *Heidi* | 0 | 0 | / | 4 | 0 |
|  | *Konrad* | 0 | 1 | / | 2 | 1 |
|  | *Moneypenny* | 0 | 0 | / | 1 | 3 |
|  | *Muppet* | 0 | 2 | / | 1 | 1 |
|  | *Pipin* | 0 | 2 | / | 0 | 2 |
| **TT 1** |  | **Wheel missing condition** | | | | |
|  | *Dolittle* | 1 | 1 | 0 | / | 2 |
|  | *Heidi* | 2 | 1 | 0 | / | 1 |
|  | *Konrad* | 1 | 0 | 1 | / | 2 |
|  | *Moneypenny* | 0 | 0 | 1 | / | 3 |
|  | *Muppet* | 0 | 0 | 0 | / | 4 |
|  | *Pipin* | 0 | 0 | 0 | / | 4 |
| **TT 2** | *Doliittle* | 1 | 0 | 0 | / | 3 |
|  | *Heidi* | 1 | 0 | 2 | / | 1 |
|  | *Konrad* | 2 | 0 | 2 | / | 0 |
|  | *Moneypenny* | 3 | 0 | 0 | / | 1 |
|  | *Muppet* | 2 | 0 | 2 | / | 0 |
|  | *Pipin* | 1 | 3 | 0 | / | 0 |

**Table S5.** **Detailed individual data for TT3 & TT4 for each subject.**

Detailed individual data: lock touched first within the four trials of each condition (depending on lock missing) for TT3 & TT4 for each individual subject. The correct response is highlighted in grey, and significant departures from chance are bold framed. Note that in TT4 non-functional locks can still be touched and removed and therefore incorrectly chosen.

| **Transfer** | **Subjects** | **First touched** | | | | |
| --- | --- | --- | --- | --- | --- | --- |
| **TT 3**  **Condition 1**  **Pin & bolt**  **missing** |  | *Pin* | *Screw* | *Bolt* | *Wheel* | *Bar* |
|  | *Doliittle* | / |  | / | 3 | 1 |
|  | *Heidi* | / | 3 | / | 1 | 0 |
|  | *Konrad* | not motivated | | | | |
|  | *Moneypenny* | / | 1 | / | 2 | 1 |
|  | *Muppet* | / | 0 | / | 3 | 1 |
|  | *Pipin* | / | 1 | / | 1 | 2 |
| **TT 3**  **Condition 2**  **wheel & screw**  **missing** | *Doliittle* | 1 | / | 0 | / | 3 |
|  | *Heidi* | 0 | / | 0 | / | 4 |
|  | *Konrad* | not motivated | | | | |
|  | *Moneypenny* | 0 | / | 0 | / | 4 |
|  | *Muppet* | 0 | / | 0 | / | 4 |
|  | *Pipin* | 0 | / | 0 | / | 4 |
| **TT 4**  **Condition 1**  **wheel**  **non-functional** | *Doliittle* | 0 | 0 | 0 | n.f. | 4 |
|  | *Heidi* | 2 | 1 | 1 | n.f. | 0 |
|  | *Konrad* | not motivated | | | | |
|  | *Moneypenny* | 0 | 0 | 1 | n.f. | 3 |
|  | *Muppet* | 0 | 1 | 1 | n.f. | 2 |
|  | *Pipin* | 1 |  |  | n.f. | 3 |
| **TT 4**  **Condition 2**  **screw**  **nonfunctional** | *Dolittle* | 0 | n.f. | 0 | 1 | 3 |
|  | *Heidi* | 1 | 3 | 0 | 0 | 0 |
|  | *Konrad* | not motivated | | | | |
|  | *Moneypenny* | 0 | n.f. | 1 |  | 3 |
|  | *Muppet* | 0 | 1 | 0 | 0 | 3 |
|  | *Pipin* | 0 | n.f. | 1 | 0 | 3 |

***3. Detailed account of individual’s trajectory during task acquisition***

We present below details of each individual’s history during acquisition, organized by acquisition group. The presence of large individual differences makes this information potentially relevant for future interpretation of the results. For the total experience with each constellation see Table S6.

*Individual Simultaneous*

In the IndSim group only the subadult male, **Pipin** removed all locks without requiring extra trials within the time given. Pipin was also the only subject who used his foot instead of his beak to remove the screw (see Movie S1).

The juvenile female **Heidi** also received the entire configuration and first succeeded in opening the screw (L4) four sessions after having removed the pin (L5) from the second session onwards. She, however, failed to open the entire configuration without additional trials and thus received stepwise and social cues (IndInc, SocInc procedures) for L1- L2, L1-L3 and L1-L4.

The juvenile female **Moneypenny** only removed the pin within the five sessions while all locks were still present. She also mastered the extra incremental trials (IndInc) with just L1 and L2 only after having received additional demonstrations by Pipin (SocInc trials). She, thereafter, succeeded in removing L3 and L4 within the frame of the incremental procedure without requiring further demonstrations.

The juvenile male **ZoZo** never passed the pre-training even after receiving demonstrations.

*Individual Incremental*

The juvenile male **Dolittle** first (after the pretraining) received two locks (L1 & L2), which he opened after one unsuccessful session. He thereafter stopped opening L2 again for five sessions and therefore received four extra trials (SocInc) with demonstrations from Konrad before becoming consistently successful in opening both locks. From then on he reliably and consistently opened all further locks (L3, L4 and L5) the first time they were presented to him.

The juvenile male **Konrad** immediately opened L1, L2 &L3 the first time they were presented (first L1, L1-2; then L1-3) and continued to do so in all following trials. Once L4 (the screw) was added, he remained unsuccessful. Only after demonstrations by Pipin did Konrad remove the screw for the first time. He was able to repeat the previous actions in all following trials and also reliably removed L5 upon first presentation.

*Social Simultaneous*

The subadult male **Figaro** first received the entire configuration and demonstrations by Pipin before every trial but removed only L5 (the pin) within the time given. When subsequently presented with extra social demonstrations in an incremental manner he reliably opened all locks up to L4 (first L1-L2; then L1-L3). He, however, failed five consecutive extra SocInc sessions with four locks (L1-L4) and was therefore removed from the test.

The juvenile male **Muppet,** who was tested under the same initial conditions (with Konrad as demonstrator) also removed only L5 for five sessions, and therefore received extra SocInc trials before every session. Within the frame of the SocInc procedure he solved all remaining locks.

*Social Incremental*

The juvenile male **Kiwi** received demonstrations from Konrad before every session. He never passed the pre-training.

The juvenile female **Mayday** received demonstrations from Dolittle. She passed the pre-training in the first session. She succeeded also in the next step (L1& L2), but never finished an entire session (of ten trials) in doing so; in total she opened the two locks three times within ten sessions before she was removed from the test.

**Table S6.** **Number of sessions with the constellations (L1-LX) until first successful opening of the entire constellation.**

Number of sessions with the constellations (L1-LX) until first successful opening of the entire constellation (D= How many sessions of that total number were preceded by demonstrations; NS= not solved). Note: this does not refer to the individual locks: for example L1-L5 does not mean that it took the subject the given number of sessions to solve L5, but that the subject had received the constellation L1-L5 that many times fore solving the entire constellation (it may already have removed parts of it eg. L5 and L4 before).

| ***Group*** | ***Subject*** | **No. of sessions with each lock until first solving it** | | | | |
| --- | --- | --- | --- | --- | --- | --- |
|  |  | ***L1*** | ***L1-L2*** | ***L1-L3*** | ***L1-L4*** | ***L1-L5*** |
| **IndSim** | *Pipin* | 3 | 1 | 1 | 1 | 5 |
|  | *Heidi* | 5 | 6 (1D) | 10 (5D) | 8(3D) | 8 |
|  | *Moneypenny* | 7 (2D) | 7(2D) | 1 | 3 | 7 |
|  | *ZoZo* | NS | | | | |
| **IndInc** | *Dolittle* | 5 | 2 | 1 | 1 | 1 |
|  | *Konrad* | 1 | 1 | 1 | 9(4D) | 1 |
| **SocSim** | *Figaro* | 2D | 1D | 1D | NS | |
|  | *Muppet* | 5D | 1D | 1D | 5D | 8D |
| **SocInc** | *Kiwi* | NS | | | | |
|  | *Mayday* | 1D | 2D | NS | | |
